# Large-Scale Multi-UAV Task Allocation via a Centrality-Driven Load-Aware Adaptive Consensus Bundle Algorithm for Biomimetic Swarm Coordination

**DOI:** 10.3390/biomimetics11010069

**Published:** 2026-01-14

**Authors:** Weifei Gan, Hongxuan Xu, Yunwei Bai, Xin Zhou, Wangyu Wu, Xiaofei Du

**Affiliations:** 1 School of Computer and Information Engineering, Institute for Artificial Intelligence, Shanghai Polytechnic University, Shanghai 201209, China; 2 School of Computer and Information Engineering, Shanghai Polytechnic University, Shanghai 201209, China; 3 School of Computer Science, University of Liverpool, Liverpool L69 3DR, UK; 4 School of Mechanical and Electrical Engineering, Harbin Engineering University, Harbin 150001, China

**Keywords:** large multi-UAV task allocation, heterogeneous UAV systems, consensus-based bundle algorithm, centrality-driven clustering, load-aware cluster self-regulation, K-medoids

## Abstract

Large multi-UAV mission systems operate over time-varying communication graphs with heterogeneous platforms, where classical distributed task assignment may incur excessive message passing and suboptimal task–resource matching. To address these challenges, this paper proposes CLAC-CBBA (Centrality-Driven and Load-Aware Adaptive Clustering CBBA), an enhanced variant of the Consensus-Based Bundle Algorithm (CBBA) for large heterogeneous swarms. The proposed method is biomimetic in the sense that it integrates swarm-inspired self-organization and load-aware self-regulation to improve scalability and robustness, resembling decentralized role emergence and negative-feedback workload balancing in natural swarms. Specifically, CLAC-CBBA first identifies key nodes via a centrality-based adaptive cluster-reconfiguration mechanism (CenCluster) and partitions the network into cooperation domains to reduce redundant communication. It then applies a load-aware cluster self-regulation mechanism (LCSR), which combines resource attributes and spatial information, uses K-medoids clustering, and triggers split/merge reconfiguration based on real-time load imbalance. CBBA bidding is executed locally within clusters, while anchors and cluster representatives synchronize winners/bids to ensure globally consistent, conflict-free assignments. Simulations across diverse network densities and swarm sizes show that CLAC-CBBA reduces communication overhead and runtime while improving total task score compared with CBBA and several advanced variants, with statistically significant gains. These results demonstrate that CLAC-CBBA is scalable and robust for large-scale heterogeneous UAV task allocation.

## 1. Introduction

Unmanned Aerial Vehicles (UAVs) have been increasingly deployed across a spectrum of civilian applications—including logistics, communications support, and disaster response—capitalizing on their inherent agility, self-organizing networks, and rapid deployment. Concurrently, they fulfill critical military functions such as precision strikes, persistent surveillance, and electronic warfare actions [1,2,3]. In rapidly varying, tightly constrained environments, multi-UAV formations demonstrate distinct advantages; their decentralized architecture and inherent flexibility facilitate the management of complex missions and enable scalable cooperative behaviors [4,5]. Consequently, task allocation emerges as a cornerstone of system performance, directly governing collaborative efficiency, communications overhead, and ultimate mission success. This critical role firmly establishes it as a principal research frontier within multi-agent systems and aerial robotics [6].

Task allocation represents a fundamental computational barrier in scaling unmanned systems, especially as mission complexity escalates with fleet size in large-scale UAV operations [7]. Optimal assignment critically governs mission tempo, operational precision, resource utilization, and spectrum efficiency. The inherent complexity stems from multidimensional constraints: aligning heterogeneous platform capabilities with diverse mission requirements while simultaneously addressing dynamic priorities and strict temporal limitations [8]. This intricate balance positions the development of robust allocation methodologies as both a crucial research priority and an enduring technical challenge in dynamic multi-agent environments.

Multi-UAV mission coordination strategies are structurally organized into centralized and distributed computing paradigms [9]. Hierarchical control architectures employ a primary computing node for comprehensive task optimization, demonstrating viability in restricted operational scopes while suffering from computational intractability and structural fragility in large-scale dynamic deployments [10]. Conventional implementations typically frame the resource allocation problem through mathematical programming constructs including multi-agent path planning (mTSP) [11,12,13], fleet routing optimization (VRP) [14], discrete constraint mixed-integer optimization formulations (MILP) [15], and time-varying network flow models (DNFO) [16], with resolution generally relying on stochastic optimization techniques [17]. While achieving operational competence in stable laboratory conditions, their architectural dependence on unified control creates critical vulnerabilities in contested operational domains featuring communication degradation or component fragility, presenting fundamental limitations in system resilience, spectral efficiency, and environmental adaptation. Conversely, distributed autonomous systems execute task assignment through localized consensus-building and coordinated decision processes, offering enhanced architectural flexibility. These peer-to-peer coordination mechanisms substantially improve operational survivability and dynamic performance metrics, though they impose significantly more demanding latency and reliability requirements on wireless networking subsystems.

Distributed algorithms have become a major focus of recent studies because they fit naturally with large, dynamically changing systems. These algorithms usually employ iterative optimization processes to achieve efficient resource allocation while satisfying operational constraints. Nevertheless, classical realizations based on the contract-net protocol [18] or auction and market rules [19] often suffer from poor efficiency and unstable behavior when the environment changes rapidly. While Markov Decision Process (MDP) frameworks [20] and Model Predictive Control (MPC) strategies [21] can achieve global coordination, they often suffer from slow convergence rates. To address these limitations, consensus-based task allocation mechanisms have emerged as prominent solutions. The Consensus-Based Bundle Algorithm (CBBA) demonstrates particular promise through its decentralized architecture, exhibiting notable scalability and robustness. By integrating local task bundling, utility assessment, and distributed agreement phases, CBBA is able to generate fast and coherent task assignment in environments characterized by evolving objectives or unstable communication networks [22,23].

While CBBA has made substantial progress in resolving distributed coordination issues, significant limitations persist in large-scale formations and heterogeneous mission environments. Two fundamental challenges emerge from current implementations. First, scalability remains limited: as operational networks expand both in vehicle count and task diversity, accumulating communication overhead and channel contention increase system latency and impair convergence performance [24]. Second, many CBBA realizations rely on relatively homogeneous platform assumptions and do not fully capture practical variations in payload capacity, operational endurance, and sensor suites [25]. This oversight can lead to systematic resource allocation imbalance [26], where some units approach saturation while others remain underutilized, reducing overall efficiency and increasing mission risk.

From a biomimetics perspective, large-scale multi-UAV coordination shares key characteristics with natural swarms: individuals interact locally over time-varying connectivity, yet coherent global behaviors emerge without centralized control. Two recurring principles in such systems are (i) self-organization, where macroscopic structures (e.g., clusters, backbones, or leader-like roles) emerge from local interactions, and (ii) load balancing via feedback, where resource pressure is redistributed through negative-feedback regulation to avoid congestion and over-exploitation. These principles provide a natural design lens for scalable task allocation under communication constraints and heterogeneous capabilities.

In this paper, the biomimetic inspiration is concretized into two implementable operators. First, a centrality-driven mechanism enables self-organization by identifying topologically influential UAVs and forming cooperation domains around them, analogous to the emergence of backbone structures in collective systems. Second, a load-aware self-regulation mechanism implements a negative-feedback loop: it monitors load imbalance and triggers split/merge reconfiguration to redistribute resource pressure, analogous to how natural swarms adaptively reallocate agents/paths when imbalance is detected.

Motivated by time-varying communication graphs and heterogeneous resources in large multi-UAV teams, and drawing on swarm-inspired self-organization and load-aware self-regulation mechanisms, we propose a Centrality-Driven and Load-Aware Adaptive Clustering CBBA (CLAC-CBBA) framework. The proposed framework first partitions the global network into cooperative domains through a Centrality-based Adaptive Cluster-Reconfiguration Mechanism (CenCluster). Within each domain, it employs a Load-aware Cluster Self-Regulation Mechanism (LCSR) to construct multi-cluster structures, dynamically adjusting cluster configurations using real-time load feedback to maintain balanced resource utilization. This hierarchical architecture supports local CBBA bidding and communication within clusters, followed by phased conflict resolution among cluster heads and key nodes, ultimately achieving globally consistent task allocation. Simulation results show that, across scenarios with 50–200 UAVs, CLAC-CBBA significantly reduces communication overhead and improves both task completion rate and total reward compared with conventional CBBA and state-of-the-art variants. The method further maintains superior robustness and convergence efficiency under dynamic network conditions and load fluctuations, confirming its practical applicability to large-scale heterogeneous UAV systems.

This paper makes the following key contributions:

- We propose CLAC-CBBA, which integrates CenCluster and LCSR within a self-organized communication topology. This integrated design enables UAV clusters to dynamically maintain balanced group sizes and resource loads. By combining intra-cluster bidding with multi-stage synchronization among clusters and key nodes, CLAC-CBBA achieves fast and consistent global convergence while effectively balancing assignment efficiency and communication overhead in large-scale heterogeneous UAV networks.
- We propose a CenCluster mechanism, which enables topology-aware, decentralized grouping of UAVs. By integrating multi-index network centrality metrics and adaptive domain reconfiguration, CenCluster alleviates communication overhead and scales efficiently with network size.
- We develop an LCSR mechanism, which maintains long-term equilibrium and robustness in heterogeneous UAV networks. By integrating hybrid resource–position clustering based on the K-medoids algorithm with real-time load perception and adaptive cluster adjustment, LCSR enables continuous workload balancing and efficient resource utilization under dynamic mission conditions.

The paper proceeds with the following structure. Section 2 surveys existing algorithms and identifies research gaps in distributed task allocation. Section 3 establishes the conceptual foundations and formulation of the proposed framework. Section 4 elaborates the complete methodological workflow and implementation details. Section 5 analyzes empirical results and discusses comparative performance evaluations. The concluding section summarizes key findings and suggests promising avenues for further investigation.

## 2. Related Work

The core problem of task allocation involves determining optimal mission distribution across UAVs to maximize temporal efficiency, representing a discrete optimization paradigm. Although involving limited quantities of drones and missions, multi-UAV assignment maintains computational intractability within the NP-hard complexity class. This inherent challenge has motivated development of heuristic methodologies that generate practically viable solutions with bounded optimality guarantees. Contemporary allocation frameworks are broadly categorized into centralized and decentralized computational architectures. Centralized implementations predominantly employ mathematical programming techniques [27], graph-theoretic formulations [28], and systematic exploration methods suitable for constrained-scale aerial networks. The methodological spectrum further encompasses population-based optimization approaches—including evolutionary algorithms [29], swarm intelligence systems [30], ant colony optimization [31], and hybrid evolutionary frameworks [32]—which have demonstrated empirical capability to identify either local or global optima within predefined computational budgets.

Notwithstanding theoretical optimality guarantees, centralized architectures require dedicated control infrastructure for mission planning and distribution. While potentially achieving global optimization under ideal conditions, these approaches incur substantial computational overhead and communication demands while exhibiting constrained fault tolerance. Performance degradation becomes particularly evident in scenarios involving component failures or dynamic network connectivity.

Decentralized coordination frameworks—such as those derived from game-theoretic principles [33], and consensus-oriented decision systems [34] and auction-inspired negotiation mechanisms [35]—operate without central supervision by empowering UAVs to independently formulate mission sequences and reconcile allocation conflicts via peer-to-peer information exchange. This structural methodology optimizes mission execution efficiency, strengthens operational survivability, and significantly diminishes susceptibility to centralized component failures, ultimately establishing a fault-tolerant autonomous decision architecture.

Beyond task allocation, multi-UAV systems have received considerable research attention in distributed cooperation and motion planning methodologies. For instance, Jin et al. [36] devised a cooperative source-localization scheme for multi-rotor platforms that emphasizes formation maintenance. Their method combines consensus-based filtering with a derivative-free search procedure, allowing a group of quadrotors to locate an emission source even under limited communications, while alleviating the difficulties of gradient estimation and formation stabilization. In a different line of work, Zhou et al. [37] proposed a unified space–time trajectory planner for miniature UAV swarms operating in cluttered and previously unknown environments. The planner enables fully autonomous flight by simultaneously addressing obstacle traversal, prevention of vehicle–vehicle collisions, and coordinated group maneuvering.

Market-driven task-allocation schemes have gained particular prominence in decentralized task assignment due to their computational tractability and operational efficiency in distributed systems [38]. The integration of auction mechanisms with consensus protocols led to the development of the CBBA, which eliminates centralized auctioneers through standardized bidding procedures across UAV networks [39]. This framework demonstrates convergence to at least 50% of Nash equilibrium solutions within bounded timeframes [40], though achieving consensus necessitates substantial information exchange. Concurrently, navigation methodologies have been established with proven asymptotic optimality under infinite domain expansion [41]. Asynchronous conflict resolution protocols minimize redundant transmissions [42]; Hierarchical consensus architectures enable scalable solutions through distributed planning layers [43]. Specialized implementations include cluster-first strategies for emergency response scenarios [44] and bid-filtering extensions that enhance communication efficiency despite potential reassignment difficulties [45]. Grouped CBBA (G-CBBA) further optimizes coordination through preference-based organization [8], though its effectiveness diminishes in communication-constrained environments with stochastic target distributions and unreliable bid synchronization.

Sophisticated mission design enhances operational realism through carefully structured task configurations and advanced decision architectures. Coordinated multi-UAV operations demand synchronized execution, especially when utilizing specialized equipment or heterogeneous platforms. Research by Nayak et al. [46] has examined asynchronous CBBA performance in partially-connected networks, revealing limitations in managing complex mission interdependencies. To address these constraints, the Task Coupling Constraint extension (CBBA-TCC) was developed for search and rescue applications [9], introducing an additional consensus phase to coordinate heterogeneous teams and interdependent tasks. In related work, Wang et al. [47] proposed a Consensus-Based Timetable Algorithm (CBTA) that refines the order and timing of task activation in decentralized teams. By shortening the start times of individual tasks, their method improves temporal coordination and, in turn, boosts overall mission efficiency. These algorithmic innovations collectively advance solution quality and computational performance in complex multi-agent operational environments.

A heuristic computational framework termed Performance Impact (PI) was established by Zhao et al. [48], enabling parallelized task evaluation and assignment updates under constrained local communications. Subsequent refinement by Whitebook et al. [49] incorporated a stochastic selection operator to augment global search capabilities and circumvent suboptimal convergence. Turner [50] advanced this lineage through the PI-MaxAss protocol, implementing temporal slot generation for unallocated missions to maximize assignment completeness. For dynamic operational environments, Yang et al. [51] devised a distributed reallocation methodology utilizing subteam coordination and progressive task liberation, ensuring conflict-free reassignment with minimal communication overhead. The EEPI mechanism introduced by Wang et al. [52] further elevated mission success probabilities without computationally expensive rescheduling. Notwithstanding these advances, the recently proposed dual-layer CBBA architecture [53] remains constrained by predetermined grouping strategies, lacking the essential capability for real-time cluster reconfiguration according to fluctuating communication demands, thereby impeding the critical evolution from static to dynamically adaptive organizational structures.

Although existing studies have substantially advanced task-allocation methods for multi-UAV teams, several issues are still unresolved when the fleet size and mission complexity grow. Typical difficulties include heavy message exchange in time-varying networks, inadequate handling of platform heterogeneity, and poor scalability in tightly coupled task settings. Motivated by these gaps, we develop a CLAC-CBBA that combines the CenCluster module with the LCSR mechanism. By introducing dynamic cluster formation and real-time load feedback regulation within a hierarchical CBBA architecture, the proposed algorithm achieves comprehensive performance improvements across communication efficiency, task execution quality, temporal effectiveness, heterogeneous resource utilization, and system scalability, as detailed in Section 4.

## 3. Modeling of the Task Assignment Problem

### 3.1. Problem Formulation

The study examines a distributed mission–assignment scenario for a heterogeneous UAV fleet. Each UAV is equipped with unique resource capabilities and can only perform certain categories of tasks within predefined time windows. The UAV fleet is defined as

(1) $$U=\left\{U_{1},U_{2},\ldots ,U_{N_{u}}\right\}=\left\{U_{1}^{\left(\alpha \right)},\ldots ,U_{n_{\alpha }}^{\left(\alpha \right)},U_{n_{\alpha }+1}^{\left(\beta \right)},\ldots ,U_{n_{\alpha }+n_{\beta }}^{\left(\beta \right)}\right\}$$

where $U_{i}$ denotes the *i*-th UAV in the fleet and *i* is the UAV index. The quantities $n_{\alpha }$ and $n_{\beta }$ indicate how many UAVs belong to classes α and β, respectively, and thus $N_{u}=n_{\alpha }+n_{\beta }$.

The mission set is denoted by T and expressed as

(2) $$T=\left\{T_{1},T_{2},\ldots ,T_{N_{t}}\right\}=\left\{T_{1}^{\left(\gamma \right)},\ldots ,T_{n_{\gamma }}^{\left(\gamma \right)},T_{n_{\gamma }+1}^{\left(\delta \right)},\ldots ,T_{n_{\gamma }+n_{\delta }}^{\left(\delta \right)}\right\}$$

where $T_{j}$ denotes the *j*-th task and *j* is the task index. Here, $n_{\gamma }$ and $n_{\delta }$ are the numbers of type-γ and type-δ tasks, yielding $N_{t}=n_{\gamma }+n_{\delta }$ tasks in total.

For notational convenience, define the index sets $I=\{1,\ldots ,N_{u}\}$ and $J=\{1,\ldots ,N_{t}\}$. The overall objective is to determine a binary assignment matrix $X={\left[x_{ij}\right]}_{N_{u}\times N_{t}}$ that maximizes the aggregated reward under UAV load and task constraints:

(3) $$\max_{X}\sum_{i\in I}\sum_{j\in J}r_{ij}\;x_{ij}$$

subject to

(4) $$\sum_{j\in J}x_{ij}\leq L_{i},\;\forall i\in I$$

(5) $$\sum_{i\in I}x_{ij}=d_{j},\;\forall j\in J$$

(6) $$\sum_{i\in I}\sum_{j\in J}x_{ij}\leq \min\;\left(N_{t},\sum_{i\in I}L_{i}\right)$$

(7) $$\mathrm{TW}\left(T_{j}\right)=\left[\sigma_{j}^{s},\sigma_{j}^{e}\right],\;\forall j\in J.$$

Here, $r_{ij}$ denotes the expected reward achieved when assigning task $T_{j}$ to UAV $U_{i}$, and $x_{ij}\in \left\{0,1\right\}$ is a binary decision variable that equals 1 if task $T_{j}$ is assigned to UAV $U_{i}$ and 0 otherwise. The parameter $L_{i}$ represents the maximum load (task capacity) of UAV $U_{i}$, $d_{j}$ denotes the required number of UAVs to complete task $T_{j}$, and $\mathrm{TW}(T_{j})$ specifies the valid execution window for task $T_{j}$.

While the model admits a binary 0–1 integer-program representation, the main challenge stems from coordinating discrete allocation actions under time-varying network connectivity, heterogeneous resources, and communication constraints, rather than from solving a single static optimization instance. Classical centralized or globally informed binary solvers scale poorly in such dynamic multi-UAV settings. To address this, TLC–CBBA combines a hierarchical grouping scheme with consensus-driven distributed coordination, enabling simultaneous adaptation of the communication topology, resource deployment, and UAV–task assignment, and consequently delivering conflict-free, near real-time allocation with improved scalability.

### 3.2. Overview of CBBA

Within CBBA, operations alternate between two core phases: the task bundle construction stage and the consensus stage. During task bundle construction, each UAV $U_{i}$ incrementally compiles its task bundle by applying a locally greedy selection mechanism aimed at maximizing individual benefit. Once this stage concludes, the consensus phase enables UAVs to communicate with neighboring agents, resolving overlapping allocations and achieving a conflict-free cooperative solution.

For each UAV $U_{i}$, a set of essential internal data elements is maintained to support cooperative decision making:

- Task bundle $\Omega_{i}$: an ordered collection of tasks currently assigned to $U_{i}$;
- Execution sequence $\Pi_{i}$: the navigation or mission order planned for $U_{i}$;
- Bid table $\Gamma_{i}$: records the maximum bids placed by $U_{i}$ over all candidate tasks;
- Allocation index $\Delta_{j}$: denotes the UAV designated to carry out task $T_{j}$;
- Communication timestamp $\Theta_{i}$: the latest time instant when $U_{i}$ successfully exchanged information with peers.

During task bundle construction, each UAV $U_{i}$ expands its bundle $\Omega_{i}$ only if adding a new task increases its cumulative payoff. In the consensus stage, UAVs exchange information with their neighbors. When UAV $U_{a}$ receives the state data from UAV $U_{b}$, it updates its internal variables according to Equation (8):

(8) $$\left\{\begin{array}{cc} \mathrm{Synchronize}: & \Gamma_{a,t}\;\leftarrow \;\Gamma_{b,t},\;\Delta_{a,t}\;\leftarrow \;\Delta_{b,t}\\ \mathrm{Reset}: & \Gamma_{a,t}\;\leftarrow \;0,\;\Delta_{a,t}\;\leftarrow \;⌀\\ \mathrm{Maintain}: & \Gamma_{a,t}\;\leftarrow \;\Gamma_{a,t},\;\Delta_{a,t}\;\leftarrow \;\Delta_{a,t}. \end{array}\right.$$

The action in Equation (8) is selected by a lookup rule based on the comparison between the received winner/bid information and the local records. Specifically, *Synchronize* is applied when UAV *b* provides a higher bid (or a more recent winner, e.g., $\Theta_{b}>\Theta_{a}$) than UAV *a*’s current record. *Reset* is applied when a conflict is detected that invalidates UAV *a*’s current bundle (i.e., a previously assumed winner is no longer valid). Otherwise, *Maintain* is applied and UAV *a* keeps its current records unchanged.

**Figure 1:**
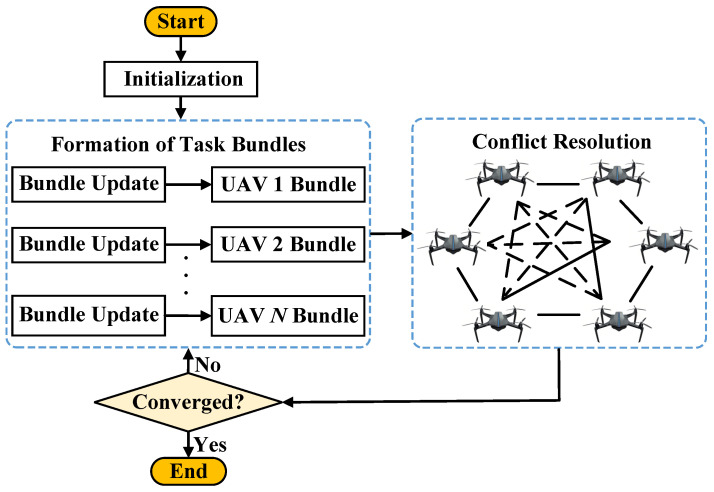
Functional workflow of CBBA. The left block illustrates bundle construction, and the right block illustrates conflict resolution/consensus. Solid links denote physical communication connectivity between UAVs, whereas dashed links indicate bid/winner information exchanges during the consensus stage (arrows show the direction of information flow).

A lookup table determines whether the agent should synchronize, reset, or maintain its local records. Upon receiving another UAV’s bid message, the recipient compares the incoming bid with its own valuation. If the received bid indicates a higher reward, the receiver updates its table and may take over the associated task. This continual bid exchange ensures consistent situational awareness and eliminates overlapping task allocations. The overall CBBA process is shown in Figure 1, where solid lines represent communication connectivity and dashed lines indicate bid/winner information exchanges during the consensus phase.

## 4. The Proposed Method

This section describes the CLAC-CBBA framework, covering the CenCluster module, the LCSR module, and implementation details of the complete algorithm.

### 4.1. A Centrality-Based Adaptive Cluster-Reconfiguration Mechanism

In large-scale heterogeneous UAV networks, achieving scalable and efficient cooperative control requires not only decentralized task coordination but also communication that is topology-aware and low in overhead. To mitigate the high communication cost of fully connected designs ($O(N^{2})$), the proposed CLAC–CBBA integrates a centrality-driven clustering procedure, termed CenCluster. For clarity, Steps 1–3 describe how anchors are identified and how UAVs are assigned to anchor domains, while Step 4 summarizes the resulting reduction in coordination/communication complexity.

**Step 1: Centrality-based group initialization.** Each UAV’s topological significance (centrality) within the current communication graph is computed via a multi-index fusion model:

(9) $$\Phi_{i}=\sum_{\kappa =1}^{4}\omega_{\kappa }\;\Phi_{i}^{\left(\kappa \right)},\;\sum_{\kappa =1}^{4}\omega_{\kappa }=1$$

where $\Phi_{i}^{\left(\kappa \right)}$ corresponds to the degree, closeness, betweenness, and eigenvector centralities of UAV *i*, and $\omega_{\kappa }$ are the corresponding fusion weights.

**Rationale and weight setting.** We fuse degree, closeness, betweenness, and eigenvector centralities because they characterize node importance in a communication graph from complementary perspectives: degree reflects local connectivity, closeness reflects global reachability, betweenness captures relay/bridge capability, and eigenvector centrality reflects influence through well-connected neighbors. Compared with relying on a single metric, this fusion provides a more robust criterion for selecting anchors (core nodes), especially under diverse and time-varying network topologies. Unless otherwise stated, we adopt equal weights $\omega_{\kappa }=0.25$ for κ=1,…,4 to avoid bias toward any single metric and to keep the method parameter-light and reproducible. The sensitivity of ω is further investigated in Section 5.5.

**Step 2: Core-node identification.** The *K* UAVs with the highest aggregated centrality $\Phi_{i}$ are selected as core nodes, serving as local communication anchors and the backbone for inter-domain coordination.

**Step 3: Topology-aware domain assignment.** All other UAVs are linked to their closest core node according to the shortest-path distance on the adjacency graph, forming *K* collaboration domains. This procedure enables dense intra-domain communication while allowing lightweight synchronization among core nodes.

**Step 4: Complexity implication.** As a direct consequence of the anchor-based partitioning in Steps 1–3, the coordination scale is reduced from $O(N^{2})$ to $O(K^{2}+NK)$, improving scalability when K≪N.

The concrete implementation of the CenCluster mechanism is given in Algorithm 1.
**Algorithm 1:** CenCluster mechanism     **Input**: Adjacency matrix A, UAV set U, centrality weights $\omega_{\kappa }$, anchor count *K*     **Output**: Final cluster domains G and anchor set K 1  Compute network centrality $\Phi_{i}$ for each UAV (Equation (9)); 2  Select the top-*K* UAVs as anchors K; 3  Assign remaining UAVs to nearest anchors based on shortest-path distance in A; 4  **return**
G and K.

### 4.2. A Load-Aware Cluster Self-Regulation Mechanism

Although topology-driven clustering enhances communication efficiency, it does not inherently ensure balanced resource utilization or workload distribution in heterogeneous UAV teams. To achieve long-term equilibrium and robustness, a LCSR mechanism is incorporated, enabling continuous refinement of the cluster layout in response to real-time system feedback.

Step 1: Hybrid resource-position clustering. Within each topological domain, UAVs are re-grouped through a hybrid similarity index that takes both functional resources and spatial proximity into account, as calculated by Equation (10).

(10) $$\Psi_{ij}=\lambda_{1}∥{\hat{r}}_{i}-{\hat{r}}_{j}{∥}_{2}+\lambda_{2}{∥{\hat{p}}_{i}-{\hat{p}}_{j}∥}_{2},\;\lambda_{1}+\lambda_{2}=1$$

where ${\hat{r}}_{i}$ indicates the normalized resource vector and ${\hat{p}}_{i}$ represents the normalized spatial position of UAV *i*. The K-medoids algorithm is employed to yield sub-clusters that are both functionally balanced and spatially cohesive.

Step 2: Real-time load estimation. During each allocation cycle, the system aggregates the task count or CBBA activity level within every sub-cluster. The degree of imbalance is defined as Equation (11).

(11) $$\Delta_{C}=\frac{\max_{j}L_{C,j}-\min_{j}L_{C,j}}{\max_{j}L_{C,j}+\varepsilon }$$

where $\Delta_{C}$ is the normalized load-imbalance index of the cluster set C, reflecting the relative deviation of workloads among its sub-clusters; $L_{C,j}$ denotes the average task load of sub-cluster *j* within cluster C; $\max_{j}L_{C,j}$ and $\min_{j}L_{C,j}$ represent the maximum and minimum workloads among all sub-clusters in C, respectively; and ε is a small positive constant introduced in the denominator to avoid division by zero and to stabilize the normalization process.

Step 3: Dynamic merging and splitting. Whenever $\Delta_{C}$ exceeds a preset threshold, the framework divides overloaded clusters or fuses underutilized ones, then re-executes the K-medoids procedure to update local memberships. This ensures that cluster size and workload remain matched in real time.

The proposed LCSR mechanism is implemented as illustrated in Algorithm 2. This process continuously reorganizes the UAV swarm in response to workload variations, maintaining balanced communication traffic and optimized resource utilization. Such dynamic adaptability overcomes the inherent limitations of static or parameter-fixed clustering approaches, enabling the CLAC-CBBA to achieve greater robustness and efficiency throughout the entire task allocation process.
**Algorithm 2:** A Load-Perception and Cluster Self-Regulation Strategy
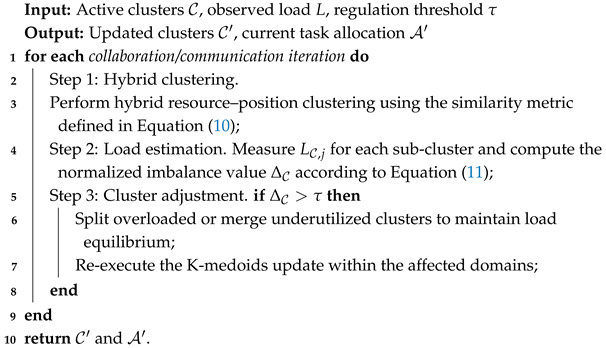


### 4.3. Implementation of the Centrality-Driven and Load-Aware Adaptive Clustering (CLAC-CBBA)

The proposed CLAC-CBBA integrates CenCluster mechanism, LCSR mechanism, and the distributed task-bundle coordination of CBBA into a unified consensus-based framework. All modules work in concert to deliver a scalable, reliable, and high-performance mission-assignment scheme for large heterogeneous unmanned-aircraft fleets.

After the CenCluster mechanism establishes a topology-aware network partition based on UAV centrality, multiple anchor domains are formed within the swarm. Within each domain, the LCSR mechanism is executed to maintain load balance and resource efficiency by forming K-medoids sub-clusters and performing split/merge adjustments when load imbalance is detected. Subsequently, the algorithm enters the distributed task allocation phase, in which UAVs perform CBBA-based bidding and bundle construction guided by dynamic scoring functions that integrate temporal, spatial, and load-related factors to achieve efficient and conflict-free task execution. The detailed process is described as follows.

#### 4.3.1. Distributed Bundle Construction and Task Allocation

Within each active sub-cluster, the UAVs collaboratively conduct distributed CBBA bidding and bundle generation under dynamic task-scoring rules. Through this process, each UAV independently evaluates candidate tasks, computes corresponding utilities, and iteratively updates its bundle until a locally optimal solution is reached. The detailed computation procedure and relevant symbol definitions are as follows.

(1) Basic reward function with time decay:

(12) $$R_{ij}=q_{j}\exp\;\left[-\lambda_{j}{\left(t_{ij}^{\mathrm{start}}-t_{ij}^{\min}\right)}^{+}\right]$$

where $R_{ij}$ denotes the base payoff for allocating task *j* to UAV *i* after applying time discounting; $q_{j}$ encodes the inherent importance of task *j*; $\lambda_{j}$ is an exponential discount factor that characterizes how strongly task *j* penalizes postponement of its start time; $t_{ij}^{\mathrm{start}}$ is UAV *i*’s earliest attainable start time for task *j*; $t_{ij}^{\min}$ denotes the earliest allowable start time of that task; and ${\left(\cdot \right)}^{+}=\max\left(0,\cdot \right)$ ensures that only positive delays contribute to decay. This formulation allows tasks with longer delays to yield smaller rewards, thereby prioritizing timely execution.

(2) Earliest start-time calculation:

(13) $$t_{ij}^{\mathrm{start}}=t_{i}^{\mathrm{avail}}+\frac{d_{ij}}{v_{i}}$$

where $t_{i}^{\mathrm{avail}}$ is UAV *i*’s available time after completing its previous bundle, $d_{ij}$ is the flight distance from the current position of UAV *i* to task *j*, and $v_{i}$ denotes its cruising velocity. This term estimates the soonest time at which UAV *i* can begin task *j*.

(3) Load-balancing penalty:

(14) $$\Gamma_{i}=\exp[-\alpha |B_{i}\left|\right]$$

where $\Gamma_{i}$ is a penalty coefficient for UAV *i* to discourage excessive task accumulation; α controls the strength of the penalty; and $|B_{i}|$ is the current number of tasks already in its bundle. A larger bundle size leads to a smaller $\Gamma_{i}$, favoring a balanced workload distribution.

(4) Compatibility constraint:

(15) $$I_{ij}=\left\{\begin{array}{cc} 1, & \mathrm{if}\;{\mathrm{compatibility}}_{ij}>0.5\\ 0, & \mathrm{otherwise}. \end{array}\right.$$

The binary variable $I_{ij}$ indicates whether task *j* is compatible with UAV *i* in terms of sensing, payload, or other capability requirements; a value of 1 signifies feasibility, while 0 excludes the assignment.

(5) Time-window feasibility check:

(16) $$F_{ij}=\left\{\begin{array}{cc} 1, & \mathrm{if}\;t_{ij}^{\mathrm{start}}\leq t_{j}^{\max}\\ 0, & \mathrm{otherwise}. \end{array}\right.$$

Here $F_{ij}$ is a Boolean indicator verifying temporal feasibility; $t_{j}^{\max}$ represents the latest permissible start time for task *j*. If UAV *i* cannot reach the task before its time window closes, $F_{ij}$ is set to 0.

(6) Final task-score function:

(17) $$S_{ij}=R_{ij}\cdot \Gamma_{i}\cdot I_{ij}\cdot F_{ij}$$

which synthesizes temporal reward, load-balancing factor, functional compatibility, and time-window constraints into a unified score. Higher $S_{ij}$ values denote tasks that yield greater overall benefit to UAV *i*.

Equation (17) adopts a multiplicative form to couple time urgency, load balancing, and feasibility in a transparent manner. The feasibility terms $I_{ij}$ (capability compatibility) and $F_{ij}$ (time-window feasibility) act as *hard gates*: if either constraint is violated, then $S_{ij}=0$ and the task is excluded regardless of its nominal reward. Given feasibility, the reward term $R_{ij}$ monotonically decreases with start-time delay, prioritizing urgent tasks, whereas the load-penalty term $\Gamma_{i}=\exp(-\alpha |B_{i}\left|\right)\in \left(0,1\right]$ decreases with the current bundle size, discouraging task accumulation. Their product therefore induces a principled trade-off: a heavily loaded UAV wins an additional task only when the time-sensitive reward is sufficiently high, promoting balanced allocation without sacrificing timeliness. In the limiting case α=0, $\Gamma_{i}=1$ and the mechanism reduces to purely reward-driven bidding, while larger α enforces stronger load regulation after feasibility filtering.

(7) Task-selection strategy: Each UAV independently updates its bundle by greedily selecting available tasks that maximize $S_{ij}$:

(18) $$B_{i}^{*}=\arg\max_{j\in T_{\mathrm{avail}}}S_{ij}$$

where $T_{\mathrm{avail}}$ denotes the set of currently unassigned or exchangeable tasks in the cluster. This greedy assignment iteratively constructs feasible, time-consistent, and locally optimized bundles within every sub-cluster, forming the basis for distributed CBBA consensus updates in subsequent stages.

#### 4.3.2. Hierarchical Conflict Resolution in CLAC-CBBA

In the proposed CLAC-CBBA, task conflict mitigation is organized as a multi-layer coordination framework to achieve consistent, scalable, and collision-free task assignment throughout the UAV swarm. By combining intra-level and inter-level synchronization, repeated task selections are avoided and overall consistency is maintained with minimal communication cost.

(1) Intra-cluster coordination. Inside every K-medoids cluster, the UAVs form an all-to-all local communication graph and run the usual CBBA bidding procedure. Each vehicle gradually builds its bundle according to locally evaluated rewards while sharing bid information with its neighbors. When several UAVs request an identical task, the one with the highest bid is selected to carry it out. If bids are identical, a predefined tie-breaking rule (for instance, node ID) determines ownership, enabling rapid stabilization of local allocations.
(2) Inter-cluster alignment. To eliminate duplicate allocations across clusters, medoid agents coordinate periodically to reconcile bidding outcomes. If the same task appears in several clusters, global bid comparison is performed and the UAV with the highest normalized score retains it. The remaining clusters promptly delete this task from their own candidate sets and reconstruct their internal bundles, which preserves agreement across clusters and facilitates stable convergence.
(3) Inter-group synchronization. At the highest hierarchy, anchor (core) nodes disseminate final allocation information through the communication backbone generated by first-layer clustering. This synchronization proceeds through three steps: (i) *intra-group aggregation*, where each anchor gathers final decisions from subordinate clusters; (ii) *inter-group exchange*, where anchors interact to harmonize inter-group discrepancies; and (iii) *hierarchical broadcast*, by which the confirmed task map is delivered network-wide via medoid and anchor relays. Through this layered negotiation, the CLAC–CBBA achieves a unified, conflict-free, and communication-efficient task allocation applicable to large-scale heterogeneous UAV systems.

#### 4.3.3. Engineering-Level Workflow and Iteration Scheduling

**Figure 2:**
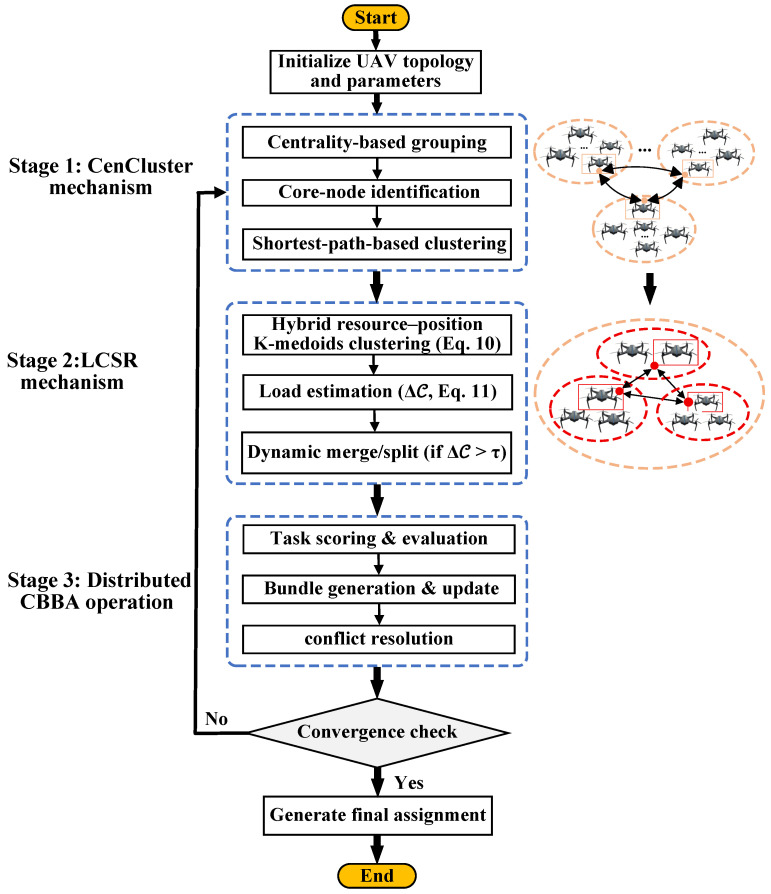
Processing pipeline of CLAC-CBBA.

For clarity and reproducibility, we summarize the engineering-level workflow of CLAC–CBBA, clarifying how clustering, bidding, synchronization, and task execution are coordinated across iterations (see Figure 2 and Algorithm 3). Each run proceeds in discrete coordination cycles t=0,1,… until convergence or $t=T_{\max}$.

(i) Initialization. Given the communication topology (adjacency matrix A), UAV states (e.g., position, remaining fuel) and task information (locations and time windows) are initialized. The initial cluster structure is generated by running CenCluster (Algorithm 1) to obtain anchor domains, followed by LCSR (Algorithm 2) to form K-medoids sub-clusters within each domain.

(ii) Cycle *t*. At the beginning of each cycle, LCSR computes the load-imbalance index (Equation (11)). If $\Delta_{C}>\tau$, the affected clusters are split/merged and K-medoids memberships are updated; otherwise the current clustering is retained. Then, *intra-cluster CBBA* is executed: within each K-medoids sub-cluster, UAVs evaluate available tasks using the score function $S_{ij}$, greedily update their bundles, and exchange local winners/bids until local consensus is reached.

Next, *inter-cluster synchronization* is performed via medoid agents, which forward summarized winners/bids to their corresponding anchor node. Anchors reconcile conflicts across sub-clusters and across anchor domains (e.g., duplicated task assignments) and update the global winners/bids. The resolved information is broadcast back to sub-clusters through anchors/medoids, and UAV bundles are updated accordingly.

(iii) State update and termination. After synchronization, the simulation updates UAV/task states according to the current allocation: completed tasks are removed, infeasible tasks (e.g., violating time windows) are discarded, and UAV resource states (e.g., remaining fuel) are updated. The algorithm terminates when winners/bids no longer change (and/or $|J^{\left(t\right)}-J^{\left(t-1\right)}|<\epsilon$) or when $t=T_{\max}$.

**Algorithm 3:** Implementation of CLAC–CBBA

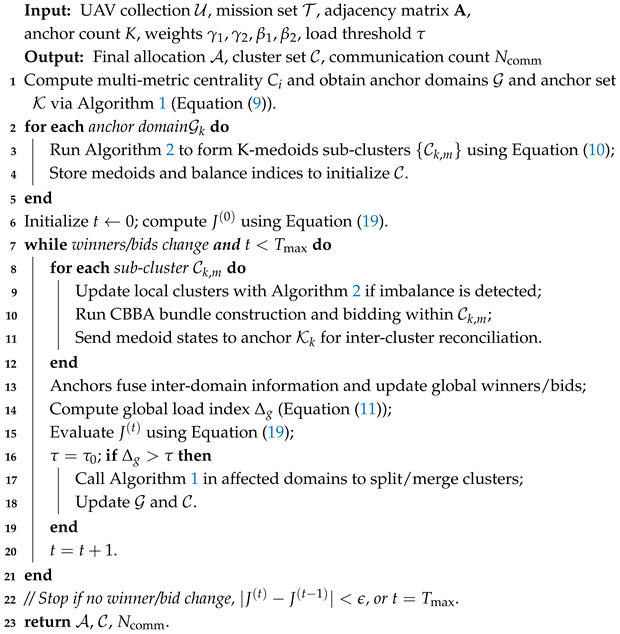



### 4.4. Overall Optimization Objective

The global objective of CLAC-CBBA seeks to maximize the overall task score (task reward) while maintaining balanced resource utilization and communication efficiency:

(19) $$\max_{A}\left\{\sum_{i\in U}\sum_{j\in T_{i}}S_{ij}-\beta_{1}\sum_{j}B_{r_{C_{j}}}-\beta_{2}\sum_{g}\Delta_{g}\right\}$$

where A denotes the final assignment, $B_{r_{C_{j}}}$ is the intra-cluster balance deviation, $\Delta_{g}$ the inter-cluster load variance, and $\beta_{1},\beta_{2}$ are weighting coefficients balancing fairness and utility.

Figure 2 and Algorithm 3 and summarize the overall execution flow of the proposed CLAC-CBBA. It integrates Cencluster, LCSR, and distributed CBBA coordination, realizing an autonomous and scalable task allocation process for heterogeneous UAV swarms.

### 4.5. Time Complexity Analysis

In the following analysis, *N* denotes the number of UAVs, *M* denotes the number of tasks, and *T* denotes the number of coordination rounds until convergence (or the maximum iteration limit). In CLAC–CBBA, *K* denotes the number of anchor domains generated by CenCluster, and $|G_{k}|$ denotes the size of anchor domain *k*, satisfying $\sum_{k=1}^{K}\left|G_{k}\right|=N$. In TLC–CBBA, *G* denotes the number of first-layer communication groups and $n_{g}$ denotes the size of group *g*, satisfying $\sum_{g=1}^{G}n_{g}=N$. Moreover, *B* denotes the maximum bundle length (or the number of candidate tasks evaluated per UAV after candidate-set truncation).

**Time complexity of CLAC–CBBA.** The per-round cost of CLAC–CBBA can be decomposed into *intra-domain computation/communication* and *inter-domain coordination*. Within each anchor domain, the LCSR K-medoids update and intra-cluster consensus interactions depend on the domain size; a conservative upper bound is $\sum_{k=1}^{K}O(|G_{k}{|}^{2})$, which accounts for pairwise distance evaluations and dense (near all-to-all) information exchanges within domains. Task scoring and bundle construction incur O(N·M) in the worst case (if each UAV evaluates all tasks), which can be reduced to O(N·B) when candidate-set truncation or a bundle-length cap is used. Inter-domain aggregation and conflict reconciliation via anchors/medoids have an upper-bounded communication cost of $O(N\cdot K+K^{2})$. Therefore, the per-round time complexity of CLAC–CBBA can be written as

(20) $$O\;\left(\sum_{k=1}^{K}{\left|G_{k}\right|}^{2}\;+\;N\cdot K\;+\;K^{2}\;+\;N\cdot M\right)$$

and the overall complexity over *T* rounds is approximately

(21) $$O\;\left(T\cdot \left(\sum_{k=1}^{K}{\left|G_{k}\right|}^{2}\;+\;N\cdot K\;+\;K^{2}\;+\;N\cdot M\right)\right).$$

When domains are approximately balanced ($|G_{k}|\;\approx N/K$), we have $\sum_{k=1}^{K}{\left|G_{k}\right|}^{2}\approx O\left(N^{2}/K\right)$. Hence, when K≪N, the dominant communication term decreases from $O(N^{2})$ to approximately $O(N^{2}/K)$, indicating improved scalability.

**Time complexity of the standard CBBA and the recent variant TLC–CBBA.** CBBA is a classical distributed task-allocation baseline. Under a fully connected or dense communication setting, winner/bid information exchange can be approximated as all-to-all, leading to a per-round communication complexity of $O(N^{2})$ and an overall complexity of $O(T\cdot N^{2})$, plus the per-round task-scoring and bundle-update cost O(N·M) (or O(N·B) with candidate truncation). As a more recent CBBA variant, TLC–CBBA adopts a two-level clustering structure: the first level forms *G* communication groups (sizes $n_{g}$), and the second level further clusters within each group and runs CBBA inside sub-clusters, while key nodes perform intra-group aggregation, inter-group exchange, and hierarchical broadcast. Under a conservative upper bound, its per-round complexity can be expressed as

(22) $$O\;\left(\sum_{g=1}^{G}n_{g}^{2}\;+\;N\;+\;G^{2}\;+\;N\cdot M\right)$$

where $\sum_{g=1}^{G}n_{g}^{2}$ accounts for dense intra-group/intra-cluster interactions, and $O(N+G^{2})$ accounts for aggregation, inter-group synchronization, and broadcast. Multiplying by *T* yields the overall complexity

(23) $$O\;\left(T\cdot \left(\sum_{g=1}^{G}n_{g}^{2}\;+\;N\;+\;G^{2}\;+\;N\cdot M\right)\right).$$

**Complexity comparison and scalability discussion.** Under full topologies, standard CBBA incurs near all-to-all winner/bid exchanges per round, leading to $O(N^{2})$ communication. TLC–CBBA [53] reduces global synchronization via two-level clustering, but still involves dense intra-group interactions $\sum_{g=1}^{G}O\left(n_{g}^{2}\right)$ and can approach $O(N^{2})$ in the worst case. In contrast, CLAC–CBBA compresses cross-domain coordination to *K* anchors and performs self-regulation within domains, yielding a dominant per-round cost of $\sum_{k=1}^{K}O(|G_{k}{|}^{2})+O\left(N\cdot K+K^{2}\right)$, which becomes approximately $O(N^{2}/K+N\cdot K+K^{2})$ under balanced domains ($|G_{k}|\approx N/K$). Hence, when K≪N, CLAC–CBBA offers improved scalability over CBBA and TLC–CBBA, consistent with the observed reductions in runtime.

## 5. Simulation Experiments

This section provides the simulation setup for cooperative mission assignment in a multi-UAV system, including the settings of UAV agents and tasks, the communication topology, the baseline algorithms used for comparison, and the main parameter choices. Next, the CenCluster component of the CLAC-CBBA scheme is examined to verify the soundness of its structural design. The influence of incorporating the LCSR mechanism is then analyzed, followed by an overall assessment of the proposed CLAC-CBBA system. Finally, the approach is quantitatively compared with several benchmark methods, and the statistical significance of the performance differences is evaluated.

### 5.1. Experimental Setup

#### 5.1.1. Scenario and Parameters

The test scenario is designed as a large-scale multi-UAV cooperative task-allocation scenario, aiming to emulate high-density swarm operations and to evaluate the scalability of the proposed algorithm under intensive communication and resource-interaction conditions. Since most existing CBBA-based studies consider fewer than 50 UAVs, this work conducts both small-scale and large-scale experiments. The small-scale setting involves 2–24 UAVs, whereas the large-scale setting uses 50–150 UAVs. For each swarm size, the fleet composition follows fixed proportions: 35% attack, 33% transport, and 32% reconnaissance UAVs. The number of tasks is set to 1.2 times the number of UAVs so as to construct heterogeneous, computation-intensive cooperative environments.

*Heterogeneity modeling and initialization.* In this work, UAV heterogeneity is modeled primarily at the decision-making level through (i) type-dependent capability/resource profiles (e.g., payload limit and task-handling capacity) and (ii) task-feasibility/compatibility constraints used in the CBBA scoring and bundle construction. To isolate the effect of the proposed hierarchical consensus and adaptive clustering mechanism on scalability and communication efficiency, we adopt a unified platform model across UAV types in the simulations, i.e., all UAVs share the same kinematic model and cruising speed, the same communication range (as specified by the adjacency graph), and the same task-execution model unless otherwise stated. Richer platform-level heterogeneity can be incorporated by assigning type-specific parameters (e.g., $v_{i}$, communication radius, and service time) without changing the proposed framework; therefore, the reported results mainly reflect the coordination and scalability benefits under controlled platform assumptions.

For each swarm size, UAV types are assigned according to the fixed proportions (35% attack, 33% transport, and 32% reconnaissance). UAVs are placed on a predefined two-dimensional lattice, and the mapping from UAV types to lattice locations is randomized while preserving the prescribed type proportions. Capability values are instantiated using predefined type-specific profiles (i.e., fixed constants per UAV type), and the same generation rule is used across all swarm sizes to ensure comparability.

At the beginning of each run, all UAVs are positioned on the predefined two-dimensional lattice. Unless otherwise stated, each platform can handle at most 10 tasks and starts with 1000 units of fuel. Tasks are randomly distributed in an 800×800×800 three-dimensional operational space, which induces frequent interactions among clusters. (Unless otherwise stated, UAVs are initialized on the 2D lattice at a fixed altitude, and task locations are specified in 3D.)

Each task is associated with a time window: the release time is drawn from [0,1000], and the duration lies between 50 and 200 time units (so that the latest feasible start time is determined accordingly). Unless noted otherwise, these simulation settings are kept identical across all experiments.

*Practical mission-profile relevance.* The considered setting is inspired by a representative mixed-mission UAV operation, where heterogeneous platforms (reconnaissance, attack, and transport UAVs) collaboratively execute geographically distributed tasks under time-window constraints and limited resources. Such profiles commonly arise in real deployments including area surveillance and reconnaissance, time-critical inspection/engagement, and emergency delivery/supply missions. The simulated task time windows emulate real-world temporal constraints and coordination pressure in these operations.

The CLAC–CBBA scheme is tested on several communication topologies—complete (fully connected), tree, dense, star, ring, random, and sparse-random graphs—to systematically examine its robustness, scalability, and coordination efficiency in large-scale operations.

#### 5.1.2. Benchmark Algorithms and Parameter Configuration

The CLAC–CBBA is assessed in detail by comparing it against Clustering–Clustering- CBBA [8], DMCHBA [27], the standard CBBA [34], G-CBBA [43], and TLC-CBBA [53]. For statistical reliability, each experimental configuration is executed 30 times under independent Monte Carlo repetitions.

In CLAC–CBBA, the key parameters $\gamma_{1}$ and $\gamma_{2}$ are both fixed at 0.5, following Bi [53]. The task-weight factor is set to $w_{k}=0.25$, and the fairness coefficients are set to $\beta_{1}=\beta_{2}=0.5$. The load-imbalance threshold is set to $\tau_{0}=0.30$ unless otherwise stated, guided by the sensitivity study reported in Section 5.5. Baseline algorithms adopt the parameter values recommended in their original publications, whereas the main settings of CLAC–CBBA are listed under the corresponding equations and further examined in Section 5.5.

All experiments are executed on a Windows 11 Pro machine (Microsoft Corporation, Redmond, WA, USA) equipped with an Intel Core i5-10600 CPU (Intel Corporation, Santa Clara, CA, USA) and eight NVIDIA RTX 3060 GPUs (NVIDIA Corporation, Santa Clara, CA, USA), and the implementation is based on Python 3.9.10 and PyTorch 1.9.1.

### 5.2. Evaluation of the CenCluster Mechanism Within CLAC-CBBA

This subsection investigates the effectiveness of the CenCluster mechanism. The study considers three UAV categories together with six typical communication topologies for comparison. A modified version of the algorithm that retains only the CenCluster module (denoted as CLAC-CBBA-I) is compared with the traditional CBBA. The comparison focuses on evaluating the average number of communication interactions, analyzing the necessity of core-node selection, and observing how different numbers of core nodes affect the overall communication load.

**Figure 3:**
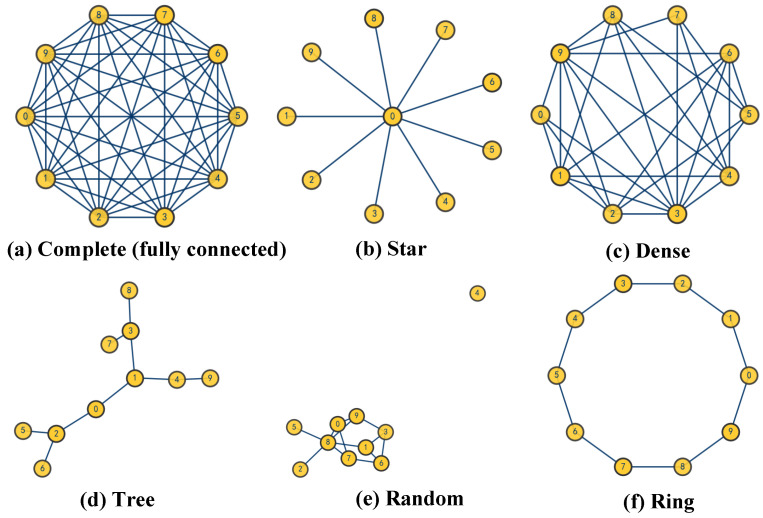
Six typical communication network structures involving ten UAVs.

Figure 3 presents examples of six representative communication topologies. In addition, Table 1 lists the IDs of the selected core nodes under different communication structures. The results show that most of the selected core nodes remain consistent across various topologies, indicating that the CenCluster mechanism can stably identify key communication hubs within the network topology and ensure that CLAC-CBBA generates consistent task-allocation schemes under different network configurations.

During the experience, it was observed that the standard CBBA failed to converge once the swarm size exceeded 150 UAVs. This phenomenon mainly results from the quadratic escalation of communication complexity with swarm scale and the frequent task conflicts that lead to information congestion. To obtain a clearer understanding of the CenCluster mechanism’s behavior, additional validation experiments are carried out in a smaller and more stable environment consisting of 50 UAVs. These experiments further investigated the number of communication exchanges under different core-node quantities and network topologies. The initial task positions were kept identical so that CLAC-CBBA-I and the baseline CBBA could achieve the same optimal task-allocation solution.

**Figure 4:**
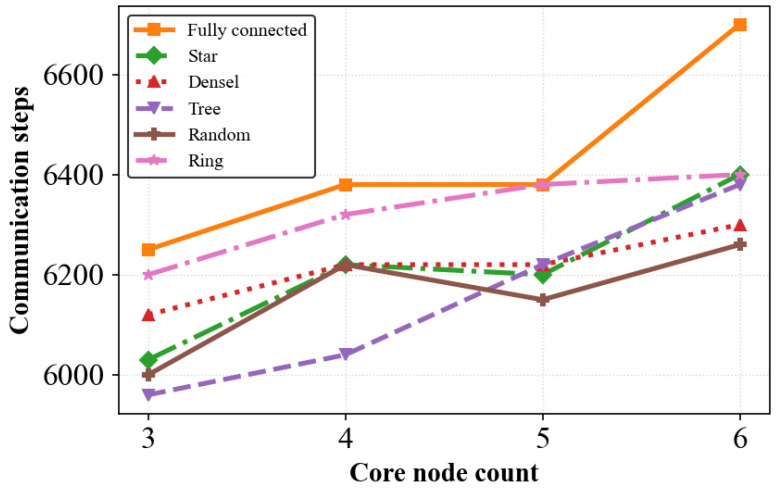
Variation of CLAC-CBBA-I communication steps with core node count across various network topologies.

Figure 4 depicts how the core-node count *K* affects the communication steps of CLAC–CBBA-I under different network topologies. Overall, increasing *K* tends to increase the communication overhead, because more anchor domains are formed and more inter-domain reconciliation among anchors is required. However, the curves are not strictly monotonic with respect to *K*. When *K* changes (e.g., from 4 to 5), the anchor set is updated by a discrete top-*K* selection, which may alter the anchor locations and the induced domain partition discontinuously. In addition, *K* controls a trade-off between intra-domain communication density and inter-domain synchronization/aggregation overhead. As a result, depending on the topology (especially for sparse or structured graphs), local decreases or increases in communication steps can occur.

**Figure 5:**
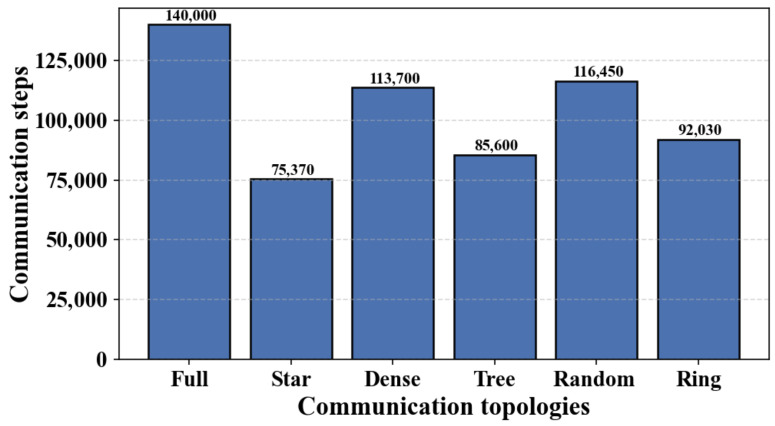
Communication load of baseline CBBA across various topologies.

Figure 5 reports the communication frequency of the original CBBA for various network structures when the core-node count is kept at five. Taken together, Figure 4 and Figure 5 show that, under the same settings, CLAC-CBBA-I yields markedly lower communication overhead than the standard CBBA. This performance improvement benefits from the CenCluster mechanism, in which cross-domain message passing is confined to core nodes only, which markedly lowers the total communication overhead.

### 5.3. Evaluation of the LCSR Mechanism Within CLAC-CBBA

**Figure 6:**
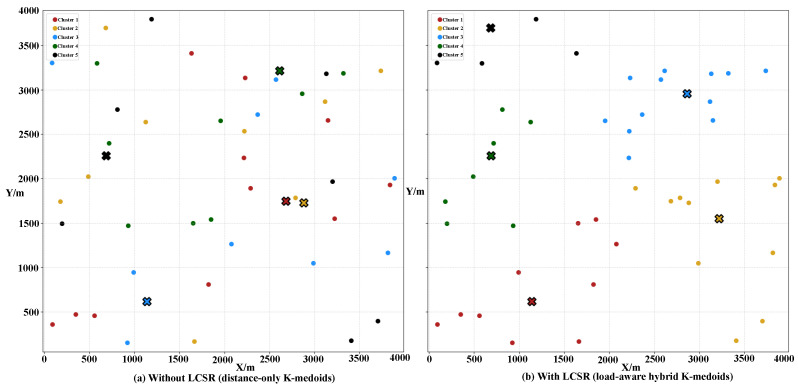
Cluster distribution before and after LCSR self-regulation: (**a**) Without LCSR (distance-only K-medoids); (**b**) With LCSR (load-aware hybrid K-medoids). Different colors represent distinct clusters of UAVs; each dot denotes a UAV, and each ‘×’ marker indicates the selected cluster medoid (the representative UAV).

Figure 6, also configured with 50 UAVs, compares the clustering results before and after applying LCSR. The distance-only K-medoids in Figure 6a leads to highly unbalanced clusters in terms of both spatial extent and cluster size, with some clusters being significantly larger than others. After introducing LCSR, as shown in Figure 6b, the clusters become more compact and comparable in size, and the medoid locations are adjusted accordingly. This results in a more balanced load distribution among clusters and yields a topology that is more favorable for subsequent task scheduling and resource allocation.

**Figure 7:**
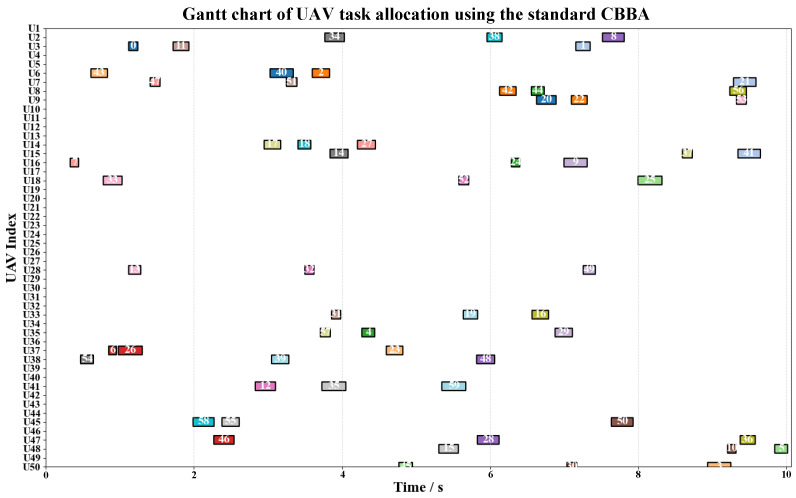
Gantt chart of UAV task allocation using the standard CBBA (Without LCSR).

**Figure 8:**
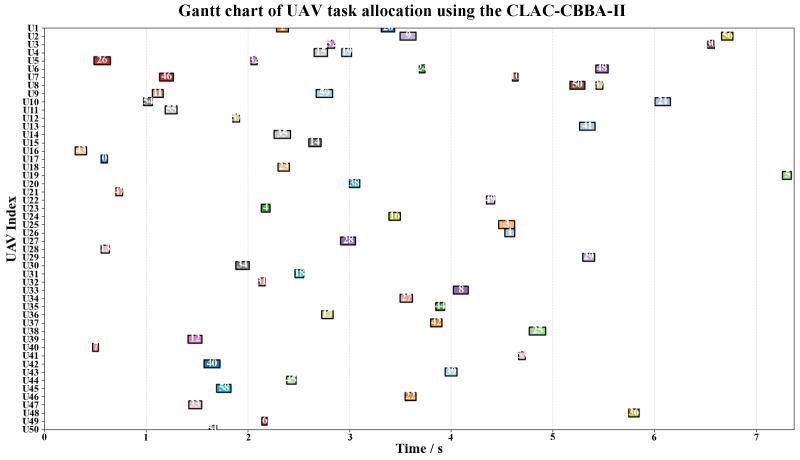
Gantt chart of UAV task allocation using the CLAC-CBBA-II (With LCSR).

Figure 7 and Figure 8 present the Gantt charts of UAV task allocation obtained with the standard CBBA (without LCSR) and the proposed CLAC–CBBA–II (with LCSR), respectively. Under the same task set, the two methods exhibit markedly different allocation patterns along both the time axis and the UAV index. As shown in Figure 7, the standard CBBA performs bidding solely based on local rewards, which leads to an unbalanced distribution of tasks among the UAVs: some UAVs (e.g., U6 and U47) execute multiple consecutive tasks, whereas many others (e.g., U10–U13, U19–U26, U29–U31, and U42–U43) remain idle for long periods. This imbalance not only increases the maneuvering and energy burden on a few UAVs but also reduces system robustness and resource utilization efficiency. In contrast, Figure 8 demonstrates that, after incorporating LCSR, CLAC–CBBA–II substantially alleviates these issues. More UAVs are simultaneously active in each time interval, tasks are distributed more evenly across the fleet, heavily loaded UAVs are significantly reduced, and the temporal coverage of tasks becomes more continuous with fewer idle gaps. These observations indicate that LCSR effectively enhances load balancing and parallelism in the task allocation process.

By explicitly accounting for cluster-level workload and UAV availability, LCSR enables CLAC–CBBA–II to maintain the overall task completion rate while achieving higher resource utilization and improved system robustness, which is consistent with the load-balancing properties observed in the clustering stage.

### 5.4. Performance Comparison and Analysis of CLAC-CBBA and Other Benchmark Algorithms

To comprehensively evaluate the performance of the proposed TLC-CBBA algorithm, two sets of experiments are conducted under small-scale and large-scale UAV scenarios. The results are compared with those of other representative algorithms in terms of communication steps, total task scores, and CPU runtime. For a clearer analysis and to minimize the effect of communication topology, a fully connected network is used, where each UAV directly exchanges information with all others, providing ideal communication conditions and allowing the evaluation to focus on the algorithm’s intrinsic performance.

#### 5.4.1. Performance Evaluation in Small-Scale UAV Scenarios

In this section, the experiments are conducted using networks containing up to 24 UAVs to investigate how the proposed algorithms behave in compact communication settings. Multiple network configurations with distinct connection densities are considered, and the density is computed according to Equation (24). For every configuration, the average number of communication rounds, the cumulative task reward, and the CPU time for a single execution are evaluated.

(24) $$\rho_{c}=\frac{\sum_{i=1}^{n}\sum_{j=1}^{n}e_{ij}}{n^{2}}.$$

Here, $\rho_{c}$ denotes the communication density of the UAV network, representing the ratio of existing communication links to all possible links. The variable $e_{ij}$ is an element of the adjacency matrix, taking the value 1 when a direct communication link exists between UAV *i* and UAV *j*, and 0 otherwise. The parameter *n* refers to the total number of UAV nodes in the system. Accordingly, a higher $\rho_{c}$ value implies a more densely connected communication network.

**Figure 9:**
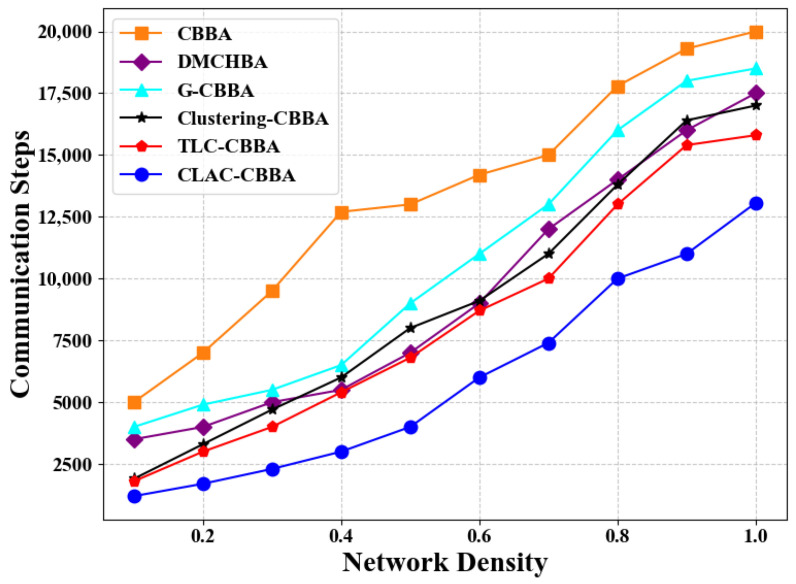
CLAC-CBBA vs. other algorithms: communication step trends across network densities (24 UAVs).

**Figure 10:**
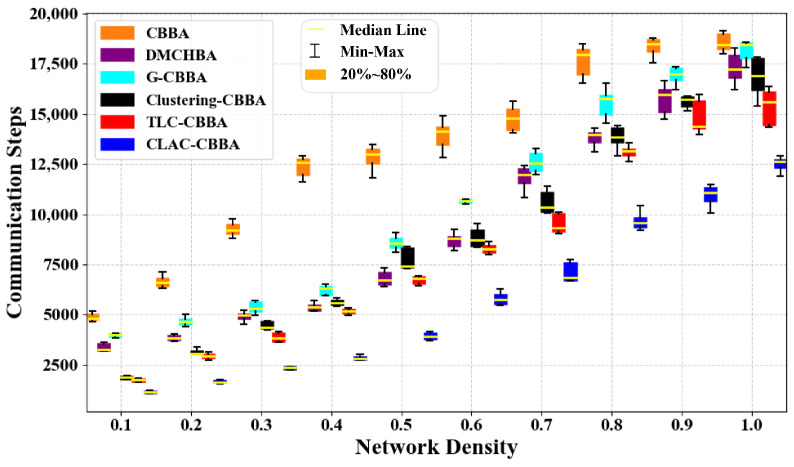
CLAC-CBBA vs. other algorithms: box-plot results across network densities (24 UAVs).

Figure 9 illustrates the differences in average communication steps between CLAC- CBBA and five comparison algorithms under various network density conditions. The results show that CLAC-CBBA requires notably fewer communication steps than the others. Figure 10 presents the corresponding box-plot analysis, where CLAC-CBBA exhibits superior performance in median and extreme values, along with a smaller interquartile range, indicating greater stability and communication efficiency.

**Figure 11:**
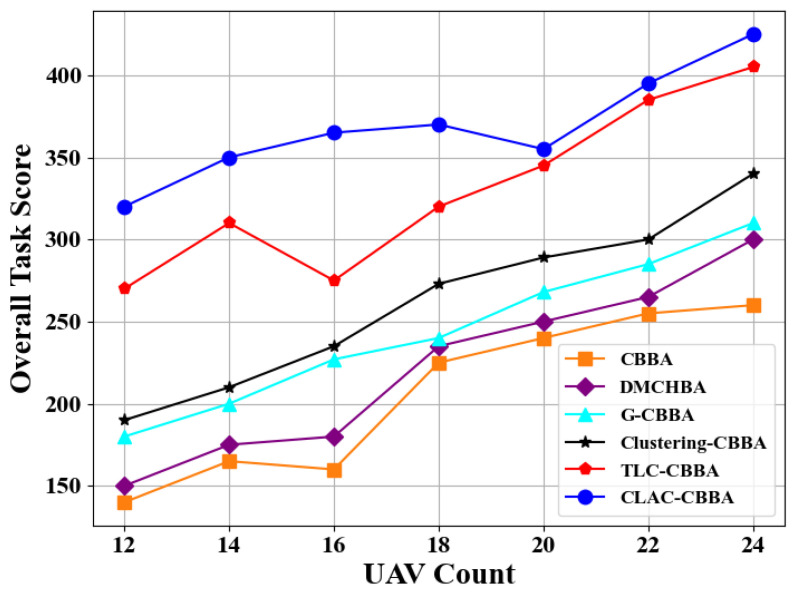
CLAC-CBBA vs. other algorithms: overall task scores in small-scale UAV scenarios.

In addition, experiments are conducted in a fully connected network with different swarm sizes (12, 14, 16, 18, 20, 22, and 24 UAVs). The objective is to evaluate the overall task performance of CLAC-CBBA under varying UAV scales and to benchmark it against four comparison algorithms. As shown in Figure 11, CLAC-CBBA consistently achieves higher total scores across all swarm sizes, with an average improvement of about 30% over the advanced methods TLC-CBBA and Clustering-CBBA. Table 2 reports detailed statistical results and comparative analysis for the 24-UAV scenario, where bold entries denote the best values. The experimental outcomes show that CLAC-CBBA consistently surpasses the four comparison methods in mean, maximum, and minimum objective values, processor running time, and confidence-interval width.

#### 5.4.2. Performance Evaluation in Large-Scale UAV Scenarios

In this large-scale experiment, a network consisting of 100 UAVs is constructed to evaluate the performance of the proposed algorithm in a compact communication environment. The evaluation focuses on three metrics: the average number of communication steps, the cumulative task reward, and the CPU time required for a single run.

**Figure 12:**
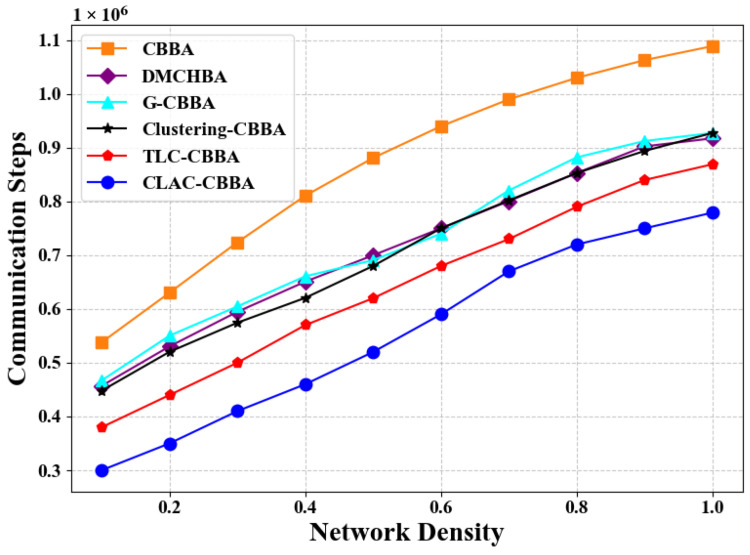
CLAC-CBBA vs. other algorithms: communication step trends across network densities (100 UAVs).

**Figure 13:**
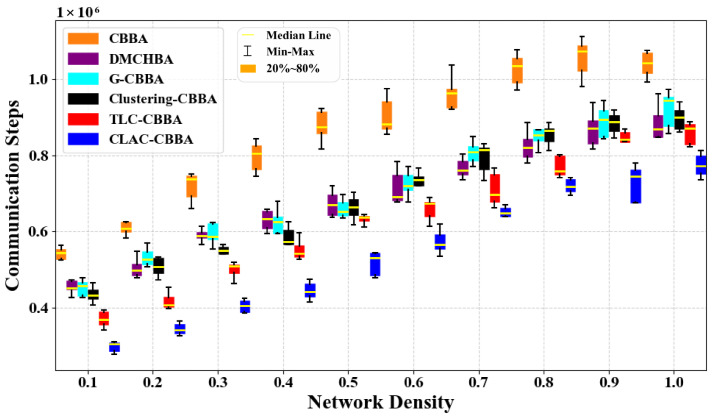
CLAC-CBBA vs. other algorithms: box-plot results across network densities (100 UAVs).

Figure 12 compares the average number of communication steps achieved by CLAC-CBBA and five benchmark algorithms under different network density conditions. It can be observed that, as the network becomes either denser or sparser, CLAC-CBBA consistently maintains a clear advantage across all operating conditions. The required number of communication steps is always significantly lower than that of the other algorithms, indicating that the proposed method is more efficient in reducing large-scale information exchange overhead. Moreover, Figure 13 presents the corresponding box-plot statistics. Compared with the benchmark algorithms, CLAC-CBBA not only exhibits better performance in terms of the median and the maximum/minimum values, but also shows a smaller box height (interquartile range) and fewer outliers. This demonstrates that the algorithm experiences smaller fluctuations across large-scale scenarios with different network densities and random instances, thereby offering better stability and robustness, while further confirming its improved communication efficiency.

**Figure 14:**
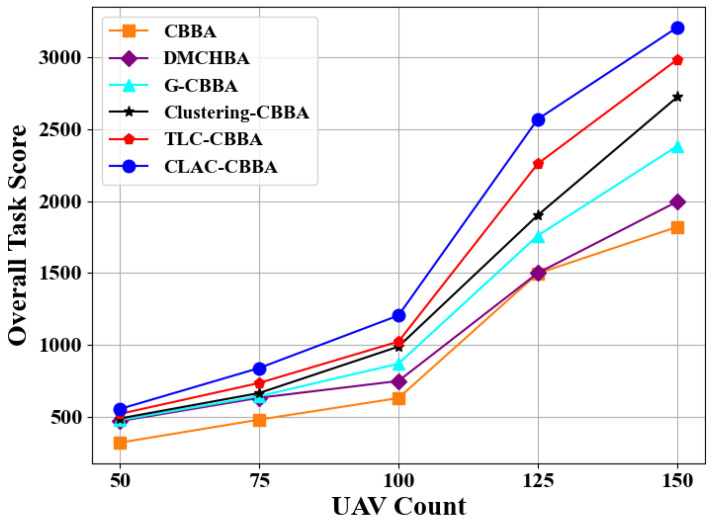
CLAC-CBBA vs. other algorithms: overall task scores in large-scale UAV scenarios.

Under the configuration where the number of tasks is set to 1.2 times the number of UAVs, experiments are run on a complete communication topology to assess the overall behavior of CLAC-CBBA for different swarm sizes (50, 75, 100, 125, and 150 UAVs) and to compare it with five alternative methods. As illustrated in Figure 14, CLAC-CBBA yields larger total rewards for every fleet size, with an average gain of about 30% relative to the advanced variants TLC-CBBA and Clustering-CBBA. Table 3 summarizes the numerical statistics for the 150-UAV case, where boldface marks the best entries. These data show that CLAC-CBBA surpasses the other five algorithms in mean, maximum, and minimum scores, processor runtime, and confidence-interval width.

### 5.5. Parameter Sensitivity Analysis

To investigate how several hyperparameters in CLAC–CBBA affect its behavior, we conduct a parameter sensitivity analysis under a fully connected topology with the swarm size fixed at 150 UAVs. We sweep the trade-off coefficients $\beta_{1}$ and $\beta_{2}$ from 0.1 to 0.9, and further examine the LCSR load-imbalance threshold τ (Equation (11)) over {0.10,0.20,0.30,0.40,0.50} while fixing $\beta_{1}=0.5$ and $\beta_{2}=0.5$. For each setting, we record the mean reward (task score), the mean number of communication iterations, and the CPU runtime.

In addition, we perform an ablation/sensitivity study on the centrality-fusion weights in Equation (9). Let the weight vector be $\omega =[\omega_{1},\omega_{2},\omega_{3},\omega_{4}]$, corresponding to degree, closeness, betweenness, and eigenvector centralities, respectively, with $\sum_{\kappa =1}^{4}\omega_{\kappa }=1$. We consider five representative configurations: the equal-weight setting A=(0.25,0.25,0.25,0.25), and four single-metric–emphasized settings, namely B=(0.40,0.20,0.20,0.20) (degree-heavy), C=(0.20,0.40,0.20,0.20) (closeness-heavy), D=(0.20,0.20,0.40,0.20) (betweenness-heavy), and E=(0.20,0.20,0.20,0.40) (eigenvector-heavy). These configurations satisfy the simplex constraint and cover moderate (non-extreme) emphasis on each metric, providing a lightweight yet informative sensitivity check.

Table 4 summarizes the results, where boldface marks the best entries. Under this experimental setting, the equal-weight configuration *A* achieves the best performance, and $\beta_{1}=0.5$ together with $\beta_{2}=0.5$ provides a favorable trade-off. Moreover, CLAC–CBBA remains stable within a reasonable range of τ, and τ=0.30 offers a good balance between load-balancing effectiveness and reconfiguration overhead. Mechanistically, τ acts as a trade-off knob between responsiveness to imbalance and the cost of split/merge reconfiguration: smaller τ triggers more frequent split/merge operations, whereas larger τ reduces the reconfiguration frequency. When the swarm size or task density increases (leading to larger and faster load fluctuations), a slightly smaller τ is often preferable; for lighter workloads, a larger τ is usually sufficient. Based on our sensitivity results, we recommend selecting τ∈[0.20,0.50] for scenarios of similar scale and load definition. Therefore, unless otherwise stated, we set $\tau_{0}=0.30$ as the default throughout this paper.

### 5.6. Statistical Significance Analysis of CLAC-CBBA

To quantify the performance gap between the proposed CLAC–CBBA and the competing algorithms, a Wilcoxon rank–sum test with a significance level of 0.05 is applied to the average task scores obtained over 30 independent runs. The test is carried out in a fully connected network with 150 UAVs. The numerical outcomes are listed in Table 5. We treat p<0.05 as evidence of a statistically meaningful difference and p>0.05 as indicating no significant deviation. To describe the strength of the effect, we also report effect sizes: values in (0,0.2] are viewed as small, (0.2,0.5] as moderate, and (0.5,0.8] as large. In the table, the symbols “+”, “–”, and “≈” indicate that CLAC–CBBA performs significantly better, significantly worse, or is statistically indistinguishable from the compared method, respectively.

The results show that CLAC–CBBA achieves consistently higher task scores than all five baselines. Specifically, the effect sizes for the comparisons with CBBA, G-CBBA, and DMCHBA fall in the range 0.82–0.87, which is typically classified as a very strong effect. For Clustering-CBBA, the value is 0.62123, still belonging to the large-effect category. and the value of 0.47346 relative to TLC–CBBA falls into the medium–effect range. These observations confirm that CLAC–CBBA offers a clear and robust advantage in task–score performance.

Overall, the experimental study demonstrates that CLAC–CBBA is highly effective for large-scale UAV task allocation. It surpasses the competing approaches in communication overhead, runtime, total task reward, and statistical significance, while maintaining stable and efficient behavior under different task sets and problem scales, thereby highlighting its strong generality and robustness.

## 6. Conclusions

This paper focuses on task allocation in large multi-UAV networks with time-varying communication links and diverse onboard resources. To cope with these difficulties, inspired by biomimetic principles of self-organization and load balancing in natural swarms, we develop CLAC-CBBA, which augments classical CBBA with CenCluster and LCSR modules to achieve efficient, robust, and scalable task assignment in complex environments. CenCluster organizes UAVs according to network-centrality measures, selects important relay nodes, and builds cooperative groups around them. In this way, redundant message passing is suppressed, key communication nodes are protected from overload, and the overall topology becomes more resilient to link variations. Within each group, LCSR employs a load-aware and position-aware K-medoids procedure to form compact clusters while redistributing heterogeneous resource loads. This design improves local coordination efficiency and strengthens the matching between tasks and available capabilities. On this basis, CBBA is then executed within each cluster to form bundles and reach local consensus, while cluster heads and central nodes periodically reconcile their decisions so that the global assignment remains consistent and free of conflicts. Numerical experiments demonstrate that CLAC-CBBA clearly surpasses the baseline CBBA and several advanced variants in terms of communication overhead, total mission reward, and runtime, and that it preserves stable behavior under a wide range of network densities, swarm sizes, and load conditions. These findings confirm that the proposed approach is effective, scales well, and is robust when applied to large heterogeneous multi-UAV formations.

Our simulations assume idealized communication and simplified execution/energy models (e.g., no packet loss or latency). In practical deployments, packet drops/delays, control/tracking errors, and energy uncertainty may affect consensus speed and allocation quality. These factors can be accommodated by extending CLAC-CBBA with time-stamped messages and timeout/retransmission, asynchronous or event-triggered updates, conservative feasibility margins, and energy-aware scoring/constraints. Future work will validate CLAC-CBBA on larger UAV swarms and more tightly coupled missions, and will explicitly incorporate unreliable links (packet loss/delay), bandwidth limits, control/tracking errors, and energy uncertainty to further improve engineering applicability.

## Figures and Tables

**Table 1:** Identification of core UAV nodes under representative communication topologies.

Typical Topology	3 Core Nodes	4 Core Nodes	5 Core Nodes	6 Core Nodes
Fully connected	5, 31, 47	5, 31, 47, 26	5, 31, 47, 26, 14	5, 31, 47, 26, 14, 39
Star	4, 31, 47	4, 31, 47, 27	4, 31, 47, 27, 15	4, 31, 47, 27, 15, 38
Dense	5, 30, 47	5, 30, 47, 26	5, 30, 47, 26, 14	5, 30, 47, 26, 14, 38
Tree	4, 30, 46	4, 30, 46, 27	4, 30, 46, 27, 15	4, 30, 46, 27, 15, 39
Random	5, 30, 47	5, 30, 47, 27	3, 30, 47, 27, 14	5, 30, 47, 27, 14, 39
Ring	3, 30, 47	3, 30, 47, 26	5, 30, 47, 26, 14	4, 30, 47, 26, 14, 38

**Table 2:** Summary statistics of total task score for the compared algorithms in the 24-UAV scenario.

Algorithms	Mean Value	Best Value	Worst Value	CI	Runtime
CBBA [34]	266	281	241	[216, 316]	4.9 s
DMCHBA [27]	301	312	287	[259, 341]	3.9 s
G-CBBA [43]	304	315	282	[269, 339]	3.4 s
Clustering-CBBA [8]	311	327	295	[281, 342]	3.3 s
TLC-CBBA [53]	405	433	387	[379, 430]	1.6 s
CLAC-CBBA	**425**	**453**	**397**	[**398**, **452**]	**1.4 s**

**Table 3:** Summary statistics of total task score for the compared algorithms in the 150-UAV scenario.

Algorithms	Average Value	Best Value	Worst Value	CI	Runtime (CPU)
CBBA [34]	1820.39	18870.52	1745.24	[1761.31, 1882.61]	308.29 s
DMCHBA [27]	1996.12	2195.32	1794.83	[1939.54, 2495.54]	292.33 s
G-CBBA [43]	2382.57	2397.41	2367.75	[2345.43, 2420.15]	263.67 s
Clustering-CBBA [8]	2723.34	2756.37	2701.29	[2694.37, 2755.72]	249.42 s
TLC-CBBA [53]	2984.23	3013.26	2783.76	[2966.55, 3012.84]	148.67 s
CLAC-CBBA	**3207.60**	**3222.45**	**3192.61**	[**3197.63**, **3217.46**]	**108.67 s**

**Table 4:** Influence of ω, $\beta_{1}$, $\beta_{2}$, and τ on the behavior of CLAC–CBBA. Bold numbers mark the best cases.

Parameter	Value	Mean Task Score	Mean Communication Steps	Runtime
Impact of ω in Equation (9) when ($\beta_{1}=\beta_{2}=0.5$, τ=0.30)				
ω	*A*	**3287.60**	**250,089**	**101.67 s**
	*B*	3200.32	254,105	111.72 s
	*C*	3195.45	251,513	114.45 s
	*D*	3201.78	251,312	110.21 s
	*E*	3196.07	251,405	113.76 s
Impact of $\beta_{1}$ when (ω = *A*, $\beta_{2}=0.5$, τ=0.30)				
$\beta_{1}$	0.10	3194.42	251,336	117.43 s
	0.30	3198.27	251,312	115.27 s
	**0.50**	**3205.57**	**251,002**	**109.63 s**
	0.70	3195.67	251,341	113.57 s
	0.90	3193.71	251,403	114.38 s
Impact of $\beta_{2}$ when (ω = *A*, $\beta_{1}=0.5$, τ=0.30)				
$\beta_{2}$	0.10	3196.47	251,418	116.32 s
	0.30	3199.86	251,327	113.93 s
	**0.50**	**3207.89**	**251,034**	**107.43 s**
	0.70	3194.58	251,352	110.37 s
	0.90	3190.63	251,451	112.68 s
Impact of τ when (ω = *A*, $\beta_{1}=0.5$, $\beta_{2}=0.5$)				
τ	0.10	3199.12	251,462	115.86 s
	0.20	3205.34	251,221	111.02 s
	**0.30**	**3208.11**	**251,045**	**108.21 s**
	0.40	3202.47	251,138	109.87 s
	0.50	3196.08	251,092	107.96 s

**Table 5:** Wilcoxon rank–sum test on task rewards for CLAC–CBBA versus the comparison algorithms.

Algorithm	*P*	Effect Size	Marker
CLAC-CBBA-CBBA	2.895243 × 10^−10^	0.848241	+
CLAC-CBBA-DMCHBA	2.882617 × 10^−10^	0.867952	+
CLAC-CBBA-G-CBBA	2.812741 × 10^−10^	0.827664	+
CLAC-CBBA-Clustering-CBBA	2.13221 × 10^−5^	0.62123	+
CLAC-CBBA-TLC-CBBA	2.16513 × 10^−2^	0.47346	+

## Data Availability

All data and information necessary to replicate the experiments, including full descriptions of the simulation setup and parameter selections, are provided in this article. Any further materials are available from the corresponding authors on reasonable request.
